# Reduced Basal Autophagy and Impaired Mitochondrial Dynamics Due to Loss of Parkinson's Disease-Associated Protein DJ-1

**DOI:** 10.1371/journal.pone.0009367

**Published:** 2010-02-23

**Authors:** Guido Krebiehl, Sabine Ruckerbauer, Lena F. Burbulla, Nicole Kieper, Brigitte Maurer, Jens Waak, Hartwig Wolburg, Zemfira Gizatullina, Frank N. Gellerich, Dirk Woitalla, Olaf Riess, Philipp J. Kahle, Tassula Proikas-Cezanne, Rejko Krüger

**Affiliations:** 1 Center of Neurology and Hertie-Institute for Clinical Brain Research, University of Tübingen, Tübingen, Germany; 2 Autophagy Laboratory, Interfaculty Institute for Cell Biology, University of Tübingen, Tübingen, Germany; 3 Graduate School of Cellular and Molecular Neuroscience, University of Tuebingen, Tübingen, Germany; 4 Institute of Pathology, University of Tübingen, Tübingen, Germany; 5 Department of Neurology, Otto von Guericke University of Magdeburg, Magdeburg, Germany; 6 KeyNeurotek Pharmaceuticals AG, ZENIT, Magdeburg, Germany; 7 Department of Neurology, St. Josef-Hospital, Ruhr-University Bochum, Bochum, Germany; 8 Department of Medical Genetics, University of Tübingen, Tübingen, Germany; Mayo Clinic, United States of America

## Abstract

**Background:**

Mitochondrial dysfunction and degradation takes a central role in current paradigms of neurodegeneration in Parkinson's disease (PD). Loss of DJ-1 function is a rare cause of familial PD. Although a critical role of DJ-1 in oxidative stress response and mitochondrial function has been recognized, the effects on mitochondrial dynamics and downstream consequences remain to be determined.

**Methodology/Principal Findings:**

Using DJ-1 loss of function cellular models from knockout (KO) mice and human carriers of the E64D mutation in the *DJ-1* gene we define a novel role of DJ-1 in the integrity of both cellular organelles, mitochondria and lysosomes. We show that loss of DJ-1 caused impaired mitochondrial respiration, increased intramitochondrial reactive oxygen species, reduced mitochondrial membrane potential and characteristic alterations of mitochondrial shape as shown by quantitative morphology. Importantly, ultrastructural imaging and subsequent detailed lysosomal activity analyses revealed reduced basal autophagic degradation and the accumulation of defective mitochondria in DJ-1 KO cells, that was linked with decreased levels of phospho-activated ERK2.

**Conclusions/Significance:**

We show that loss of DJ-1 leads to impaired autophagy and accumulation of dysfunctional mitochondria that under physiological conditions would be compensated via lysosomal clearance. Our study provides evidence for a critical role of DJ-1 in mitochondrial homeostasis by connecting basal autophagy and mitochondrial integrity in Parkinson's disease.

## Introduction

The identification of genetic causes of Parkinson's disease (PD) only ten years ago allowed first insights into the molecular mechanisms leading to neurodegeneration in this common movement disorder. These mechanisms include the pathological misfolding of disease-related proteins, disturbed ubiquitin-mediated protein degradation pathways and the accumulation of intraneuronal protein aggregates in affected brain areas, also known as Lewy bodies [1]. The identification of PD-specific mutations in nuclear genes encoding mitochondrial proteins provided the first genetic link to the variety of biochemical findings implicating a disturbed mitochondrial function in PD pathogenesis [2], [3], [4].

Mutations in the *DJ-1* gene were identified as a rare cause of autosomal-recessive PD [5] and account for approximately 1–2% of all early-onset forms of PD [6]. To date, several homozygous deletions and point mutations in the DJ-1 gene encoding a highly conserved 189 amino acid protein are known to cause PD due to a loss of protein function [5], [7], [8]. The physiological role of DJ-1 implicates broad biological functions including modulation of transcription, chaperone-like functions and antioxidant properties [9]. Although present in various subcellular compartments including cytoplasm and nucleus, targeting of DJ-1 to mitochondria was reported to contribute to its physiological cytoprotective role [10], [11]. In conditions of oxidative stress DJ-1 is converted into an acidic variant allowing it to quench reactive oxygen species (ROS) and to localize to the mitochondria [12]. Cell culture experiments revealed that oxidation of a specific cysteine residue in position 106 of the DJ-1 peptide sequence is responsible for mitochondrial targeting and protection against oxidation-induced cell death [10], [11]. Indeed, promoting the mitochondrial localization of DJ-1 increased DJ-1 dimer formation at the outer mitochondrial membrane and the cytoprotective activity towards oxidative insults [13]. *In vivo*, loss of DJ-1 function is linked to increased vulnerability to complex I inhibition as shown in fly and mouse models [14], [15].

Mitochondrial dysfunction plays a central role in neuronal cell death in PD. Impaired mitochondrial function is critically linked to imbalanced dynamic fusion and fission events of mitochondria, to energetic depression and may subsequently result in the activation of programmed cell death mechanisms [16], [17], [18]. Hence, selective removal of dysfunctional mitochondria by lysosomal degradation pathways is critical for the maintainance of cellular integrity [17], [19]. Although a critical role of DJ-1 for mitochondrial homeostasis has been recognized by different *in vitro* and *in vivo* approaches, no effects on mitochondrial dynamics and downstream lysosomal degradation pathways, such as macroautophagy (hereafter  =  autophagy) were reported. Here we provide evidence that loss of DJ-1 function causes a prominent disturbance of both, mitochondrial function and morphology, that is linked to decreased basal autophagy and impaired lysosomal degradation.

## Methods

### Cell Culture

For the functional analysis of the DJ-1 protein experiments were performed in immortalised DJ-1 knockout (KO) and DJ-1 wild-type (WT) mouse embryonic fibroblasts (MEF) that have been described previously [20]. Stably back-transfected DJ-1 MEF were generated by transfecting DJ-1 KO MEF with pcDNA3.1/Zeo (Invitrogen, USA) vector containing a DJ-1 WT construct or the empty control vector, respectively. Transfection was performed using Fugene 6 HD (Roche Diagnostics; Mannheim) according to the manufacturers' instruction.

MEF cells were cultured in a 5% CO_2_ humidified atmosphere in DMEM medium (Invitrogen, USA) containing penicillin, streptomycin (Gibco, Invitrogen, USA), and 10% fetal calf serum (FCS, Biochrom, Germany). Stable transfected DJ-1 knock-out MEF cells were cultured in DMEM medium (Invitrogen, USA) containing Zeocin (Invitrogen, USA, 400 µg/ml) and 10% fetal calf serum (FCS, Biochrom, Germany) in a 5% CO_2_ humidified atmosphere.

Moreover fibroblasts from members of the family carrying the E64D mutation in the *DJ-1* gene and a healthy control were included in our analyses [8]. Skin biopsies were taken from the index patient carrying the homozygous E64D mutation, two unaffected sibs carrying the E64D mutation in the *DJ-1* gene in a heterozygous state and a healthy age-matched control individual. The study was approved by the ethics committee of the University of Tübingen. All patients and controls gave written and informed consent. Primary fibroblast cells were maintained in RPMI medium with 10% FCS supplemented with 100 IU/ml penicillin, 100 µg/ml streptomycin and 1 mM pyruvate.

### Respirometry

Mitochondrial respiration of MEF cells (2×10^6^ cells/ml) was measured with an OROBOROS-oxygraph in Hanks solution at 30°C as described previously [21], [22]. Cell homogenates were prepared by gentle homogenisation (10^7^ cells/200 µl HBSS buffer consisting of 132 mM NaCl, 5.4 mM KCl, 0.44 mM KH_2_PO_4_, 0.34 mM NaH_2_PO_4_, 0.49 mM MgCl_2_, 0.41 mM MgSO_4_, 10 mM HEPES, 1 mM CaCl_2_, 10 mM pyruvate, pH = 7.3). Multiple substrate inhibitor titration of mitochondrial respiration was performed with cell homogenates maintained in MMMPK buffer (5 mM MgCl_2_, 120 mM mannitol, 40 mM MOPS, 5 mM KH_2_PO_4_, 60 mM KCl, pH = 7.4) as described previously [23].

### Assessment of Mitochondrial and Lysosomal Parameters by Fluorescent-Activated Cell Sorting (FACS)

Analysis of intramitochondrial reactive oxygen species (ROS) production, mitochondrial membrane potential (MMP), mitochondrial mass and lysosomal activity in DJ-1 KO MEF cells compared to DJ-1 WT MEF cells in living cells was performed by a FACS-based methods. Cells were harvested with 2 mM EDTA solution in PBS and washed with PBS. To analyze the production of intramitochondrial ROS cells were incubated with PBS containing 2 µM MitoSOX® (Invitrogen, USA) for 15 min at 37°C. The MMP was measured by staining with 200 nM tetramethylrhodaminemethylester (TMRM; Invitrogen, USA) in PBS for 15 min at 37°C. Mitochondrial mass and lysosomal activity in MEF were determined by staining the cells with either 50 nM MitoTracker® Green FM (Invitrogen, USA) or 1 µM LysoTracker® (Invitrogen, USA), respectively. To determine the relative uptake of MitoTracker® or LysoTracker® the threshold was set at about 50% of the WT signal intensity and the percentage of shift in the fluorescence activated cell sorter (FACS) staining was determined with the Summit version 4.2 software (Beckman Coulter).

For each sample, at least 30.000 cells were analyzed for the corresponding fluorescence on a CyAnTM ADP apparatus (Beckman Coulter) with a 488-nm or 415-nm laser. Experiments were performed three times with similar results.

### Immunocytochemistry and Transfection

For immunocytochemistry MEF cells were grown on collagen-coated slides and transfected with either GFP-LC3 (kindly provided by Tamotsu Yoshimori to TP-C) or the corresponding empty EGFP vector control. Cells were transfected using Lipofectamine 2000 (Invitrogen, USA) according to the manufacturer's introduction. Forty eight hours after transfection cells were washed with PBS, fixed with 4% (wt/vol) PFA in PBS for 10 min and permeabilized with 0.1% (vol/vol) Triton X-100 for 10 min. After washing with PBS cells were incubated for 30 min in 10% (vol/vol) FCS in PBS for blocking. Cells were labelled at 4°C overnight with a specific antibody against the protein of interest. The next day cells were washed with PBS and labelled with a combination of either Cy3-conjugated anti-mouse and FITC-conjugated anti-rabbit or Cy3-conjugated anti-rabbit and FITC-conjugated anti-mouse secondary antibodies (Dianova, Germany). Cell nuclei were stained with the fluorescent chromatin dye Hoechst 33342 (Molecular Probes, USA).

### Immunofluorescence Microscopy

Mitochondrial morphology was analyzed by life cell imaging. MEF cells or human fibroblasts were cultured in Lab-Tek®II chambered coverglasses (#155382; Nalge Nunc International). Mitochondria were stained with 200 nM MitoTracker® Green FM (Invitrogen, USA). Hoechst 33342 (Molecular Probes, USA) was used to stain nuclei. Analysis of mitochondrial morphology was done with an inverted Zeiss Axiovert microscope (Zeiss Plan-Apochromat 63x/1,4) with temperatured incubation chamber at 37°C. Time lapse pictures were taken with an AxioCamMRm camera (Zeiss, Germany) in 5 seconds intervals using AxioVision software (Zeiss, Germany). The series of pictures were saved uncompressed and analyzed with AxioVision software (Zeiss, Germany). For colocalization assays of mitochondria and lysosomes, additional to MitoTracker® green, the cells were stained with 200 nM Lysotracker® red (Invitrogen, USA), according to the manufacturers instructions. To determine the degree of colocalization images were loaded into Axiovision 4.7.2 software and evaluated accordingly (Zeiss, Germany).

### Analysis of Mitochondrial Morphology

Fluorescence microscopy images were optimized by adjusting the contrast and afterwards binarized by conversion to 8-bit images. After unspecific noise of the fluorescence signal was reduced, a spatial filter (convolution filter) as well as a threshold was applied to the images to define mitochondrial structures. Using Image J 1.41o software (Wayne Rasband; National Institutes of Health, USA), every single mitochondrion of the investigated cells was marked to analyze morphological characteristics such as its area, perimeter, major and minor axes. On the basis of these parameters, the aspect ratio (AR) of a mitochondrion (AR; ratio between the major and the minor axes of the ellipse equivalent to the object) and its form factor (FF; perimeter^2^/4π*area), consistent with the degree of branching, were calculated [21].

### Electron Microscopy

DJ-1 deficient and DJ-1 WT MEF were fixed in 2.5% glutaraldehyde in Hank's modified salt solution (HMSS), postfixed in 1% OsO4 in 0.1 M cacodylate buffer, scraped off, centrifuged and dehydrated in a series of ethanol. The 70% ethanol step was saturated with uranyl acetate for contrast enhancement. Dehydration was completed in propylene oxide and the specimens were embedded in Araldite (Serva, Germany). Ultrathin sections were produced on a FCR Reichert Ultracut ultramicrotome (Leica, Germany), mounted on pioloform-coated copper grids and contrasted with lead citrate. Specimens were analyzed and documented with an EM 10A electron microscope (Zeiss, Germany).

### Western Blot

Western Blot (WB) analysis were conducted as described previously [3]. Cells were lysed in buffer containing PBS, 1% Triton and protease cocktail (Roche, Germany). Lysates were separated by SDS-PAGE and analyzed by immunoblotting using antibodies against Drp1 and OPA1 (BD Transduction Laboratories, USA), hFis (Axxora, Switzerland), Mfn2 and Actin (Sigma, Germany), phospho-p42/44 MAPK (Erk1/2) (Tyr202/Tyr204; CellSignaling Technology, USA), p42/44 MAPK (Erk1/2; CellSignaling Technology, USA), Prohibitin (NeoMarkers, Fremont, CA, USA), GAPDH (Thermo Scientific, Fremont, CA, USA). Secondary antibodies were purchased from GE Healthcare (UK). On each WB 50 µg of protein were loaded per lane.

### Assessing Autophagy: GFP-WIPI-1 Puncta Formation

To conduct GFP-WIPI-1 puncta-formation analyses [22], [23], DJ-1 WT or KO MEF were transiently transfected using Lipofectamin2000 (Invitrogen, USA) at a ratio of 1∶2.5 (pEGFP.C1-WIPI-1 DNA:Lipofectamin2000) for 48 hrs according to the manufactorer's instruction. Routinely, transient transfections were controlled with empty vectors (data not shown). Autophagy was induced by incubating the cells 3 hrs (37°C, 5% CO_2_) with rapamycin (500 nM) or starvation medium (EBSS); or autophagy was inhibited by the administration of wortmannin (233 nM). For quantitative confocal microscopy of overexpressed GFP-WIPI-1, three independent prepared slides for each condition (100 cells/slide) were analyzed. Confocal microscopy was conducted using Zeiss/Axiovert 100M/LSM510 and a 63x 1.4 DIC Plan-apochromat objective [24].

### Assessing Autophagy: Endogenous LC3-II Protein Monitoring

To conduct LC3-II protein monitoring [25] of endogenous LC3 in DJ-1 WT or KO MEF, cells were lysed with hot Laemmli buffer and subjected to anti-LC3 WB analyzes using LC3 Antibody (Nanotools, Germany). LY290042 pretreatment was performed using 10 µM LY290042 for 3 hrs. Protease inhibitors (Protease inhibitor complete, Roche) were added to the culture medium and renewed every 30 min. Results were normalized over GAPDH levels or the ratio of LC3-II/LC3-I was determined based on densitometry (Image Quant 5.1).

### Mitochondrial Fractionation

Cellular fractionations were performed using a mitochondrial isolation kit for cultured cells (Pierce Rockford, USA), according to the manufacturer's instructions. The fractions received, were analyzed by Western-Blot. The mitochondrial fraction was defined using an antibody against prohibitin, the cytosolic fraction was defined by using an antibody against GAPDH.

## Results

### Decreased Respiratory Rates in Intact Mouse Embryonal Fibroblasts from DJ-1 KO Mice

**Figure 1:**
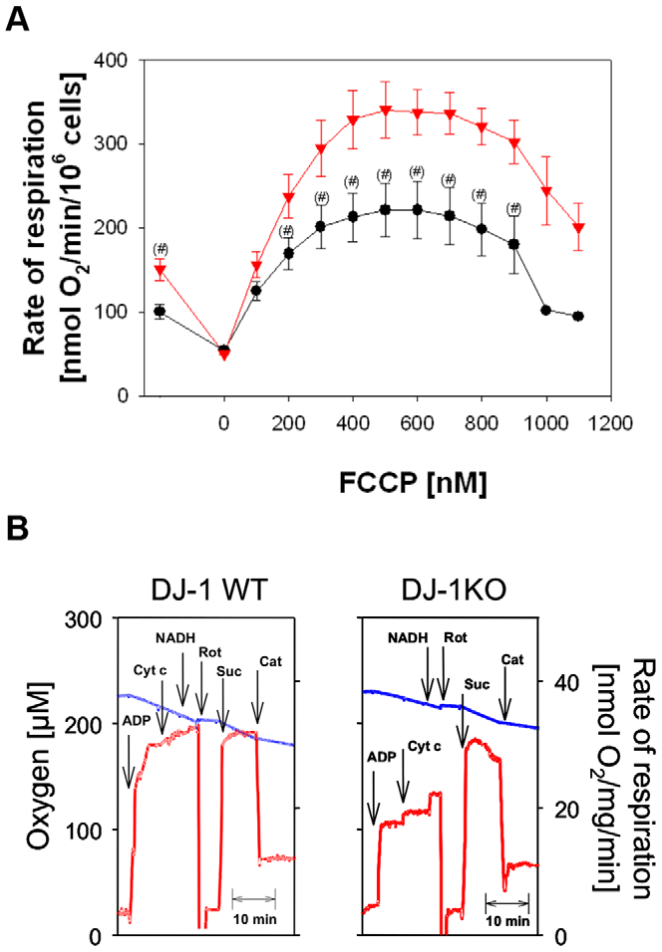
Respirometric detection of mitochondrial dysfunction in intact DJ-1 deficient and DJ-1 WT MEF cells. (A) Mitochondrial oxygen consumption was measured with high resolution respirometry in freshly harvested MEF. Cells (2×10^6^ cells/ml) were incubated in a Hank's medium containing 10 mM pyruvate. Endogenous respiration was observed without any further additions. Oligomycin resistent respiration was followed in the presence of 10 µg/ml oligomycin. Then uncoupled respiration was measured by stepwise additions of 100 nM FCCP. Data as means ± SE of 13 (wild type MEF, ▾) and 11 (KO MEF, •) single incubations performed of 5 different cell preparations. DJ-1 KO MEF showed significantly reduced oxygen consumption compared to controls (#: p<0.05, one-way ANOVA). (B) Mitochondria were investigated by respirometry in cell homogenates of DJ-1 KO cells and WT controls using multiple substrate inhibitor titration. Measurements were performed in homogenates from 3×10^6^ cells/ml MMMPK buffer with 10 mM pyruvate and 2 mM malate as substrates (Additions: ADP, 5 mM ADP; Cyt c, 10 µM cytochrome c; NADH, 150 µM NADH; Rot, 1,5 µM rotenone, Suc, 10 mM succinate; Cat, 50 µM carboxyatractylate). Blue lines indicate the oxygen concentration in the oxygraph (left ordinate), red lines represent the first derivative of the oxygen time curve, directly indicating the rate of respiration (right ordinate). Typical respirograms from four independent experiments with similar results are shown. The state 3_pyr/mal_/state 3_suc_ –ratio was 0.57±0,12 for DJ-1KO cells and 0.93±0,15% for WT cells (p<0.05).

To detect mitochondrial dysfunction by respirometry, we compared mitochondrial oxygen consumption in intact MEF cell lines from DJ-1 KO mice and littermate control cells. Measurements were performed with high resolution respirometry in freshly harvested intact cells with pyruvate as added substrate (Fig. 1A). Significantly diminished rates of endogenous respiration (−33%) were observed in DJ-1 KO cells compared to the controls. Addition of oligomycin, an inhibitor of the F_0_F_1_ATPase, allowed to measure the nonphosphorylating respiration, which was the same in both types of cells. Then titration with the uncoupler FCCP revealed a typical activation followed by an inhibition of respiration. Rates of uncoupled respiration were significantly lower (up to −34%) in DJ-1 KO compared to WT cells (Fig. 1A). These results clearly demonstrate decreased endogenous oxidative phosphorylation and diminished uncoupled respiration of DJ-1 KO cells compared to WT controls in the presence of endogenous substrates plus the added complex I-dependent substrate pyruvate.

To address whether or not specifically the complex I-dependent mitochondrial function is affected we investigated cell homogenates by means of a special substrate inhibitor titration protocol [23], [26]. This protocol allows the sequential measurement of maximum phosphorylation rates (state 3) for two different substrates in the same sample. As shown in Fig. 1B addition of 5 mM ADP increased the respiration using pyruvate and malate as substrates (state 3_pyr/mal_). However the increase in state 3 respiration was significantly lower (−50%) in DJ-1 KO cells compared to controls. After inhibition of the complex I-dependent respiration with rotenone the addition of complex II-dependent substrate succinate allowed the measurement of state 3_suc_.respiration. In both types of cells the complex II dependent respiration was comparable to each other indicating that the impairment of DJ-1 KO cells is restricted on the complex I-related mitochondrial metabolism. The ratios calculated from complex I- and complex II-dependent state 3 respiration measured in the same sample (state 3_pyr/mal_/state 3_suc_) allow the quantification of the complex I-related mitochondrial impairment [23]. Specifically state 3_pyr/mal_ respiration was significantly reduced in DJ-1 KO cells (−39%).

By additions of cytochrome c and NADH to respiring mitochondria is it possible to prove whether the permeability of outer or inner membranes for these molecules is pathologically changed [23] [26]. However, the similar and only modest increase of state 3_pyr/mal_ respiration in DJ-1 KO and WT cells (Fig. 1B) indicated intact mitochondrial membranes in both types of cells. Finally the nonphosphorylating rate of respiration was measured by inhibition of the ADN-carrier with carboxyatractyloside. As also shown for the measurements in intact cells (Fig. 1A) there was no change of nonphosphorylating rate of respiration in DJ-1 KO cells.

All these data show, that the mitochondrial impairment in DJ-1 KO cells is not caused by a general mitochondrial dysfunction but by specific impairments of complex I-dependent oxidative phosphorylation in DJ-1 KO cells which can cause energetic depression of cell metabolism [16], [18].

### Increase in Intramitochondrial ROS Formation in DJ-1 KO Cells

**Figure 2:**
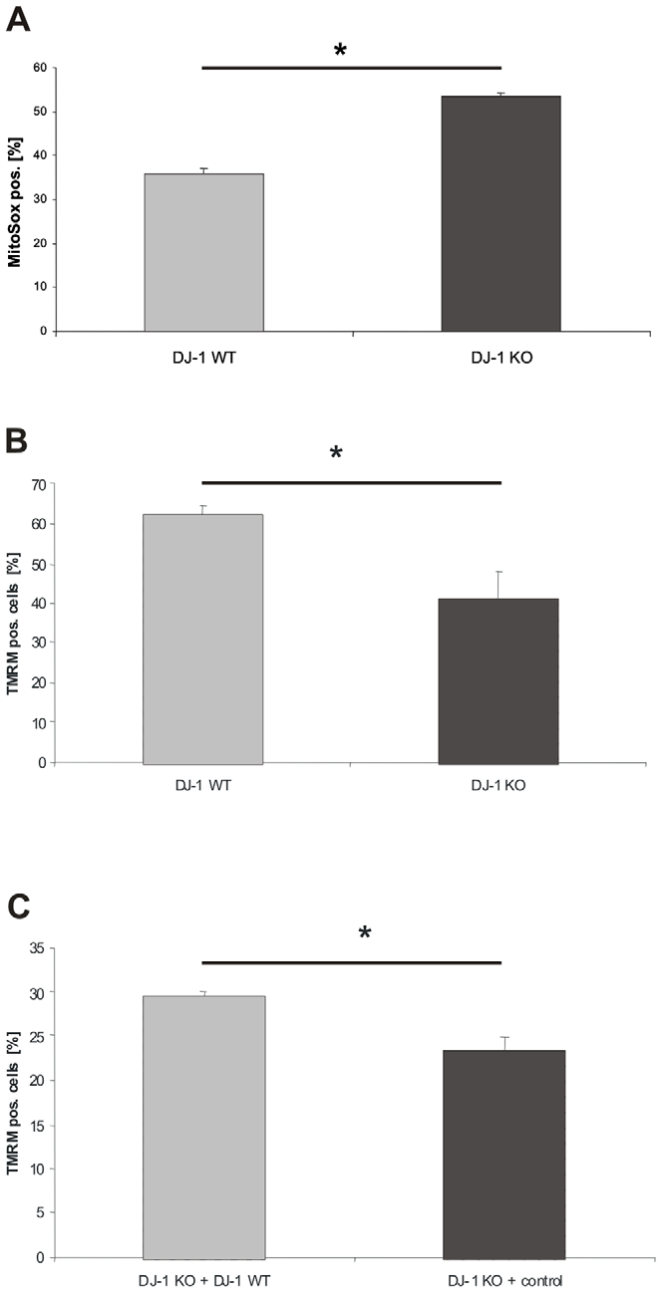
Analysis of the integrity of mitochondria in DJ-1 deficient and DJ-1 WT MEF. (A) To determine intramitochondrial ROS production, DJ-1 WT and DJ-1 KO cells were treated with MitoSox® (Invitrogen, USA) according to the manufacturer's instructions for 15 min at 37°C. Fluorescence emission was determined by FACS analysis. An increased intramitochondrial ROS production was observed in DJ-1 KO MEF compared to controls (p<0.05, Student's t-test). Mitochondrial membrane integrity was assessed in DJ-1 WT and KO MEF and in DJ-1 KO cells stably transfected with either DJ-1 WT or the corresponding empty control vector, respectively. The MMP was determined after treatment with TMRM (Invitrogen, USA) for 15 min. at 37°C. The absorption of TMRM in the mitochondria was used as a marker for the integrity of MMP and determined by FACS analysis. (B) A significantly decreased MMP was found in DJ-1 KO cells compared to controls (*p<0.001, Student's t-test). (C) Complementation of the DJ-1 KO phenotype with physiological DJ-1 significantly increased the MMP compared to empty vector control (*p<0.05, Student's t-test).

Next, we investigated how depletion of DJ-1 in our KO model interferes with mitochondrial function and integrity. One important pathophysiological property of mitochondria is the generation of ROS. Intramitochondrial ROS production was monitored and quantified by using MitoSOX Red™ (Molecular Probes, USA,), a fluorogenic dye that is targeted to the mitochondria and readily oxidized by superoxide, but not by other ROS- or reactive nitrogen species–generating systems. We found a significant increase in intramitochondrial ROS in MEF from DJ-1 KO mice compared to wild type controls (p = 0.011; Fig. 2A). Treatment of MEF with 5 mM H_2_O_2_ further increased intramitochondrial ROS production in both DJ-1 KO MEF and MEF from WT DJ-1 littermates with a higher ROS content in KO cells (data not shown). These results were replicated in 3 independently prepared MEF cell lines from either KO mice or wild type littermates. Interestingly, in the cytoplasmic compartment no increase in basal ROS was observed using H_2_DCFDA [20]. This is in line with loss of the physiological antioxidant function of DJ-1.

### Decreased MMP in MEF from DJ-1 KO Mice

Based on the alterations in oxygen consumption and increased intramitochondrial ROS production we next examined the effects of DJ-1 deficiency on the MMP as assessed by flow cytometry using TMRM (Invitrogen, USA). Analysis of DJ-1 KO MEF revealed a reduction of the MMP when compared to wild type controls, supporting an involvement of DJ-1 in mitochondrial homeostasis (p<0.001; Fig. 2B).

In order to determine whether exogenous DJ-1 would be able to rescue the observed mitochondrial dysfunction in DJ-1 KO cells, we stably transfected DJ-1 KO MEF with a plasmid expressing human DJ-1 cDNA or an empty vector control. Using TMRM staining, we found a significant increase in MMP in KO cells stably transfected with DJ-1 compared to empty vector controls (p = 0.027; Fig. 2C). These results indicate that loss of DJ-1 leads to impaired mitochondrial function that can be rescued by reintroduction of physiological DJ-1 protein into the KO background.

### Changes in Mitochondrial Morphology Due to Loss of DJ-1 Function

**Figure 3:**
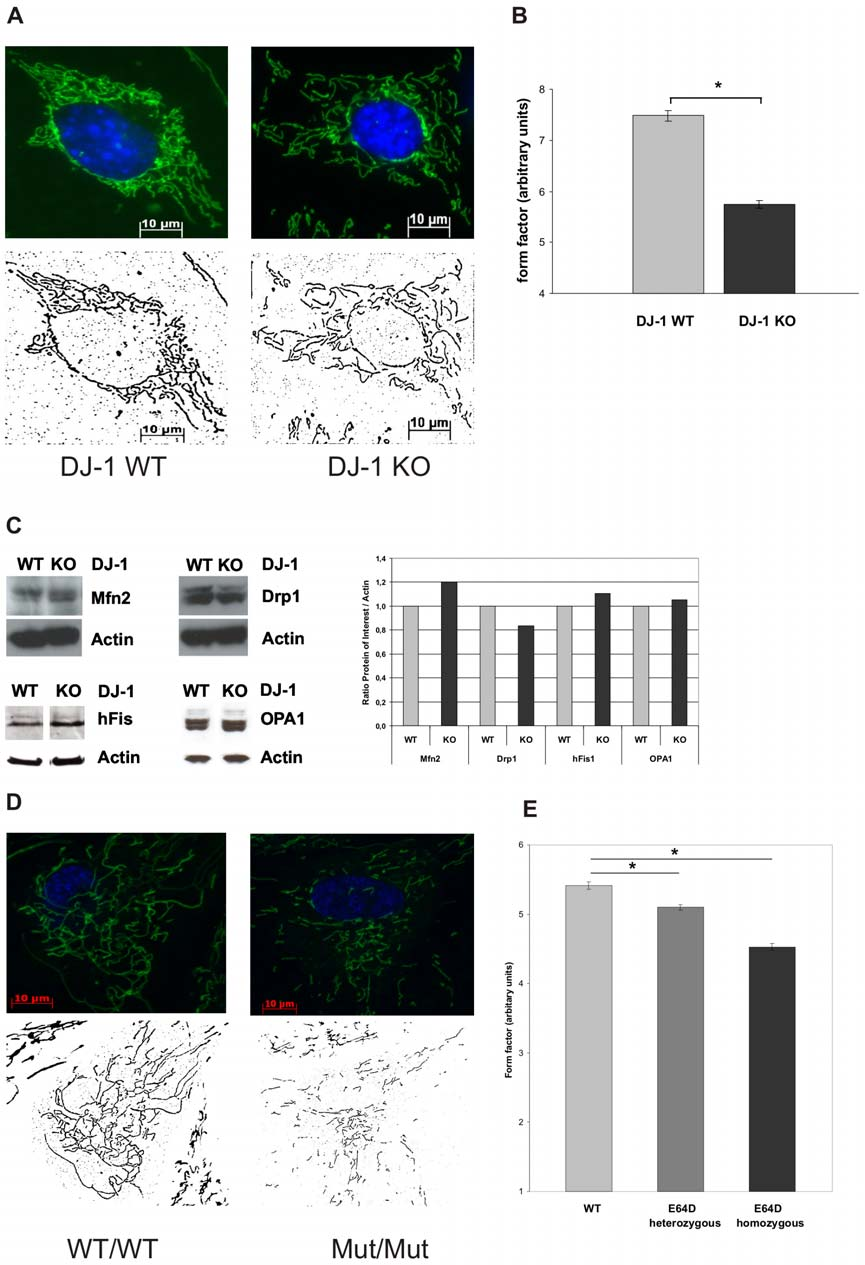
Effect of DJ-1 on mitochondrial morphology. Mitochondrial morphology in living DJ-1 KO and DJ-1 WT MEF and in human fibroblasts from carriers of the E64D mutation in the *DJ-1* gene and a healthy control were analyzed by life cell imaging microscopy (Cell Observer Z1, Zeiss, Germany) at 37°C using ApoTome®optical slides with 0.35–0.40 z-stacks. Mitochondria were stained with 200 nM MitoTracker® green FM (Invitrogen, USA), a specific mitochondrial dye, for 15 min at 37°C, nuclei were stained with Hoechst 33342 (Molecular Probes, USA; blue). (A) Fluorescence microscopy images of single mitochondria were analyzed using Image J 1.41o software (Wayne Rasband; National Institutes of Health, USA) for area, perimeter, major and minor axes. On the basis of these parameters, the aspect ratio (AR) of a mitochondrion (AR; ratio between the major and the minor axes of the ellipse equivalent to the object) and its form factor (FF; perimeter^2^/4π*area), consistent with the degree of branching, were calculated. (B) Mitochondrial branching as indicated by the form factor (FF) was significantly reduced in DJ-1 KO cells compared to WT (*p<0.001, Student's t-test). No significant differences in the AR were observed between DJ-1 KO cells and controls (not shown). Images from 45 individual cells were analyzed on three separate occasions by an investigator blinded to the experimental design. (C) Influence of DJ-1 on the expression of mitochondrial fission and fusion regulating proteins using WB analysis with specific antibodies against hFis1, OPA1, Drp1, and Mfn2. As a control for equal loading a specific antibody against β-actin was used. Densitometric quantification revealed no significant differences in the respective protein levels between WT and KO MEF. (D) Fluorescence microscopy images of single mitochondria of human fibroblasts from the E64D family and a healthy control (see supplemental figure S1) were analyzed using Image J 1.41o software (Wayne Rasband; National Institutes of Health, USA) as described previously. On the basis of these parameters, the aspect ratio (AR) of a mitochondrion (AR; ratio between the major and the minor axes of the ellipse equivalent to the object) and its form factor (FF; perimeter^2^/4π*area), consistent with the degree of branching, were calculated. (E) Mitochondrial branching as indicated by the form factor (FF) was significantly reduced in cells from the index patient carrying the homozygous E64D mutation compared to the healthy control (*p<0.001, Student's t-test). Also heterozygous carriers of the E64D mutation revealed a decreased form factor compared to control (*p<0.05, Student's t-test). Images from 40 individual cells of each proband were analyzed on three separate occasions by an investigator blinded to the experimental design.

Recent data indicate that dynamic morphological alterations of mitochondria are involved in mitochondrial dysfunctions [17]. Therefore, we applied live cell imaging technique based on MitoTracker green (Molecular Probes, USA) and concomitant Hoechst 33342 (Molecular Probes, USA) staining. In DJ-1 KO cells, we found significant differences in the mitochondrial morphology compared to WT controls. Analysis of single mitochondria revealed that DJ-1 KO cells display disintegration of the mitochondrial network and reduced branching of mitochondria. Using Image J software to assess morphological parameters as the length (aspect ratio) and degree of branching (form factor) this was reflected by significant differences in the form factor between DJ-1 KO cells and WT controls (Fig. 3A and 3B).

These results link the observed impairments of complex-I–dependent respiration, the increase in mitochondrial ROS production and the loss of MMP in DJ-1 KO cells with an unbalanced mitochondrial morphology.

Our observations imply that the mitochondrial network, which is related to dynamic changes of mitochondrial morphology, may be modulated by DJ-1. In general, the number and morphology of mitochondria is critically regulated by mediators of mitochondrial fission, such as Drp1 and Fis1, and by mediators of mitochondrial fusion, such as OPA1 and mitofusins (Mfn1 and Mfn2) [27]. Therefore, we investigated if DJ-1 leads to changes in the steady state levels of Drp1, Mfn2, Fis1, and OPA1 proteins, respectively. No differences were observed in DJ-1 KO MEF compared to wild type controls (Fig. 3C).

To control for species-specific effects, we next extended our morphological studies to human fibroblasts. Therefore we examined the mitochondrial morphology in fibroblasts from carriers of the E64D mutation in the DJ-1 gene [8]. We found that homozygosity for the mutant allele leads to a marked loss of the protein levels of DJ-1 in fibroblasts *in vivo* and thereby confirm previous *in vitro* data on the E64D and the L166P mutation that provided evidence for instability of the mutant protein (supplemental figure S1) [8]. These results indicate that fibroblasts from homozygous carriers of the E64D mutation may provide a model to investigate the consequences of loss of DJ-1 function in humans. Our analyses of human fibroblasts from the homozygous index patient of the E64D family, two sibling carrying the E64D mutation in the heterozygous state and one unrelated healthy control revealed a similar mitochondrial phenotype as observed in DJ-1 KO cells with significantly reduced mitochondrial branching in the affected index patient compared to the heterozygous carriers and the healthy control (Fig. 3D, E). Our results indicate a conserved role of DJ-1 in the regulation of mitochondrial mophology among different species.

### Impaired Lysosomal Function in DJ-1 KO Cells

**Figure 4:**
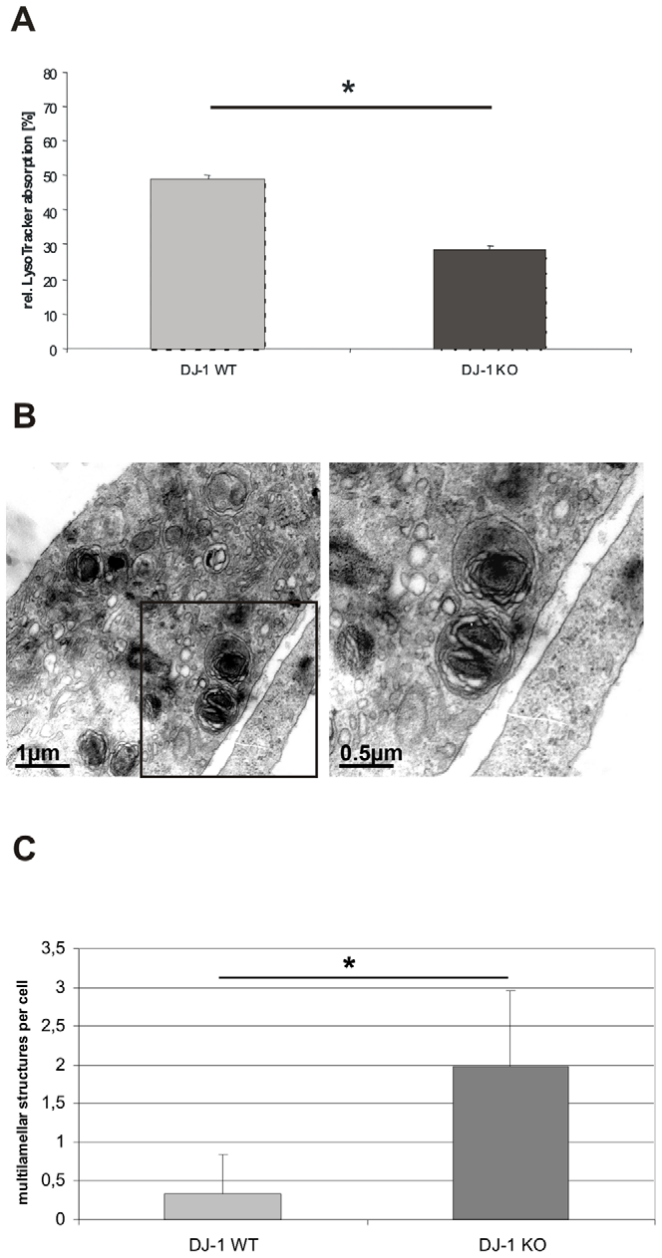
Lysosomal phenotype in DJ-1 WT and DJ-1 deficient MEF. (A) Lysosomal activity was determined by FACS analysis. Cells were stained with the specific lysosomal dye LysoTracker® (Invitrogen, USA) 1 µM for 15 min at 37°C. The absorption of LysoTracker® was measured by FACS. The uptake of LysoTracker® is an indicator of the Lysosomal mass. DJ-1 KO cells revealed a significantly reduced uptake of LysoTracker® compared to WT MEF (*p<0.0001, Student's t-test). The experiments were performed in triplicate on three different days, the diagram represents 1 of 3 sets of experiments with similar results. (B) Ultrastructural analyzes of DJ-1 deficient MEF and MEF from DJ-1 WT littermates were performed by electron microscopy. MEF lacking DJ-1 show multilamellar structures reminiscent of modified lysosomes. Bars 1 µm or 0,5 µm, respectively. (C) Quantification of lysosomal-like structures in DJ-1 WT and DJ-1 KO MEF. Multilamellar structured lysosomes were counted per cell and were significantly more frequent in DJ-1 KO cells compared to WT controls (p<0.001, Student's t-test). A total of 150 individual cells were evaluated for the presence of multilamellar structures.

Decreased MMP has been shown to interfere with mitochondrial degradation that critically relies on lysosomal function [19]. Therefore, we analyzed a potential role of DJ-1 in terms of lysosomal activity using LysoTracker® (Molecular Probes, USA) staining. We found a significant reduction in the fluorescent intensity of LysoTracker-treated DJ-1 KO MEF when compared to WT controls (Fig. 4A). These results are in line with impaired lysosomal activity and were subsequently followed by detailed ultrastructural analyzes.

Electron microscopy of DJ-1 KO MEF revealed the presence of characteristic multilamellar structures containing electron-dense material and whorled membranous material, which was only rarely seen in the corresponding controls (Fig. 4B, 4C). Interestingly, these structures were reminiscent of cellular vacuoles containing pigments associated with neurological disorders, particularly caused by lysosomal dysfunction [28], [29], [30], [31]. Indeed, autophagy-mediated lysosomal clearance is the only means by which cells degrade dysfunctional mitochondria. In this context, similar structures are believed to represent undigested cellular components that accumulate over time [31].

### Reduced Basal Autophagy in DJ-1 KO Cells

Next, we asked whether impaired lysosomal degradation pathways in the absence of DJ-1 could be proven by upstream events that depend on functional lysosomes, such as autophagic clearance. Autophagy is a lysosomal degradation pathway responsible for the degradation of cytoplasmic material including long-lived proteins, misfolded proteins and cellular organelles such as mitochondria [32], [33]. Interestingly, impaired autophagy has been correlated with the onset of neurodegeneration, such as the pathology of Alzheimer's, Huntington's and Parkinson's disease [34]. However, molecular targets that confer autophagic dysfunction in neurodegeneration are insufficiently identified. Here, we employed well established autophagy assays, such as LC3 protein monitoring [25] and WIPI-1 puncta formation quantification [22], [23], [24], [34], [35] to assess autophagy in DJ-1 KO MEF versus WT controls. Upon autophagic stimulation, LC3 becomes conjugated to phosphatidylethanolamine (LC3-II) and localizes at autophagosomal membranes. Unconjugated LC3 (LC3-I) is distributed throughout the cytoplasm, and in the nucleous. Hence the increase of endogenous LC3-II protein abundance monitors the autophagic activity. Thereby, basal autophagy as well as induced or inhibited autophagy can be compared using different cellular backgrounds. In addition, LC3 protein monitoring allows to analyze the autophagic flux, meaning the process from autophagosome formation to autophagolysosomal degradation. In experimental set-ups where autophagolysosomal degradation is blocked by the addition of lysosomal inhibitors, such as protease inhibitors, the further increase of LC3-II protein abundance reflects that autophagic flux occurs properly [36].

The WIPI-1 puncta formation analyses also monitors autophagic activity, because WIPI-1 specifically binds to Phosphatidylinositol 3-phosphate (PI(3)P) upon autophagic stimulation and localizes at autophagosomal membranes. Using GFP-WIPI-1, PI(3)P-bound GFP-WIPI-1 (puncta) can easily be visualized and quantified by fluorescence microscopy as a measure for the autophagic activity. Cells that display GFP-WIPI-1 fluorescent puncta undergo autophagic stimulation, and in cells without GFP-WIPI-1 puncta (non-puncta) autophagy is inactive. Of note, the usage of GFP-LC3 in such experiments leads to unwanted protein aggregations of GFP-LC3 that do not reflect autophagosomal membrane formation. In contrast, the usage of GFP-WIPI-1 does not lead to such protein aggregations [25].

**Figure 5:**
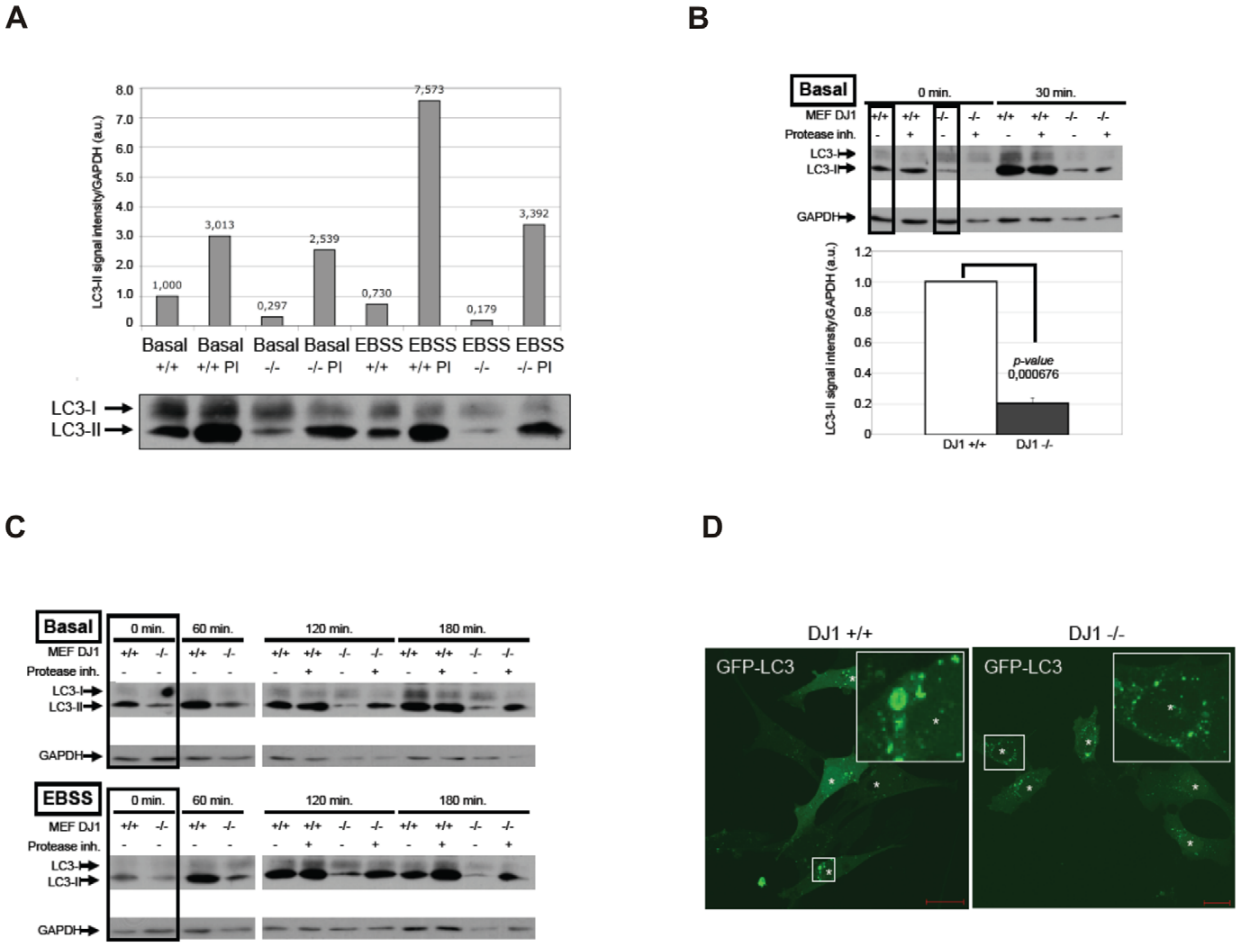
Assessing basal autophagy by LC3 protein monitoring in DJ-1 WT and DJ-1 deficient MEF. (A–C) LC3 protein monitoring reveals reduced basal autophagy in DJ-1 KO MEF. (A) WT (MEF DJ-1 +/+) or DJ-1 KO MEF (MEF DJ-1 −/−) were either left untreated (mock treatment, control) or were treated with medium lacking amino acids (EBSS) in the absence or presence of lysosomal inhibitors (PI) in the culture medium. Total protein extracts were analyzed by anti-LC3 WB. The increase of LC3-II normalized over GAPDH levels was quantified. (B, C) WT (MEF DJ-1 +/+) or DJ-1 KO MEF (MEF DJ-1 −/−) were pretreated with LY290042 for 3 hrs to reduce autophagy to a minimum basal level. Thereafter, in the presence or absence of lysosomal inhibitors (protease inh.) control medium was added to analyze basal autophagy, or EBSS was added to induce autophagy for the indicated period of time (min.). LC3 and GAPDH protein levels from total protein extracts are shown. (B) The decrease in basal autophagy is presented in the lower panel graph as follows. From three independet sets of experiments shown in C (boxed lanes in the upper and lower panels) and B (boxed lanes in the upper panel) were used to quantify the LC3-II signal intensity normalized over GAPDH. The measurements for DJ1 +/+ MEFs were set to one. The LC3-II signal intensity in DJ1 −/− MEFs was compared to DJ1 +/+ MEFs (p-value 0.000676). (D) Although basal autophagy was reduced in DJ1 −/− MEFs, autophagy was not abolished as further shown by transient expression of GFP-LC3 and detecting GFP-LC3 puncta as an indicator for autophagosomes.

Here, we first used the method of endogenous LC3 protein monitoring to ask whether or not basal autophagy is altered in models of loss of DJ-1 function. Strikingly, basal autophagy levels were reduced in DJ-1 KO MEF compared to WT control cells as reflected by a decrease LC3-II protein abundance, a measure for autophagosome-bound LC3 (Fig. 5A). We normalized LC3-II signal intensities over GAPDH in standard ECL analyses and show that the LC3-II signal intensity in DJ-1 KO cells (MEF DJ-1 −/−) was reduced to 0.297 when compared to WT cells (MEF DJ-1 +/+, set to 1) (Fig. 5A). However, the autophagic flux was not inhibited as shown by the addition of protease inhibitors (PI) during assay conditions that block final degradation (Fig. 5A). Comparing the LC3-II protein abundance during conditions with or without protease inhibitors, we found an increase of LC3-II signal intensity in WT cells from 1,000 to 3.013 (MEF DJ-1 +/+ PI) and also in DJ-1 KO cells from 0.297 (MEF DJ-1 −/−) to 2,539 (MEF DJ-1 −/− PI). This result was further verified when cells were incubated in amino acid-free medium as a treatment that induces autophagy. Here, the further increase of LC3-II signal intensities when compared to basal LC3-II intensity levels in both, WT cells (MEF DJ-1 −/− PI: 7.573) and DJ-1 KO cells (MEF DJ-1 +/+ PI: 3.392) confirm that the autophagic flux was maintained in MEF cells devoid of DJ-1 [36]. Next, we treated cells with the PI-3 kinase inhibitor compound LY294002 for 3 hours. Thereby, PI(3)P generation by Vps34/Beclin1 is inhibited and basal autophagy is reduced to a minimum. Under such circumstances, we compared basal autophagy in a time course after the LY294002 compound was removed and substituted with growth medium (Basal) or amino acid-free medium (EBSS) with or without protease inhibitors (Fig. 5B, 5C). In all cases from the three independent experiments presented in Fig. 5B (upper panel) and Fig. 5C (upper panel, lower panel), LC3-II protein abundance was reduced in the DJ-1 KO cells, but autophagic activity was not completely abolished (compare LC3-II signal intensities with and without the addition of protease inhibitors) as observed before. Normalized basal autophagy (0 min. time point in time courses from three independent experiments, boxed lanes in Fig. 5B, C) was quantified by setting the LC3-II signal intensities in DJ-1 WT cells to 1. The decrease in LC3-II signal intensity in DJ-1 KO cells was found to be significant with a *p-*value of 0.000676 (Fig. 5B, lower panel). In order to confirm that autophagy is not completely blocked, although the basal level was reduced in mutant DJ-1 cells, we transiently transfected DJ-1 WT and KO MEFs with GFP-LC3 and found puncta formation in both cell types (Fig. 5D).

**Figure 6:**
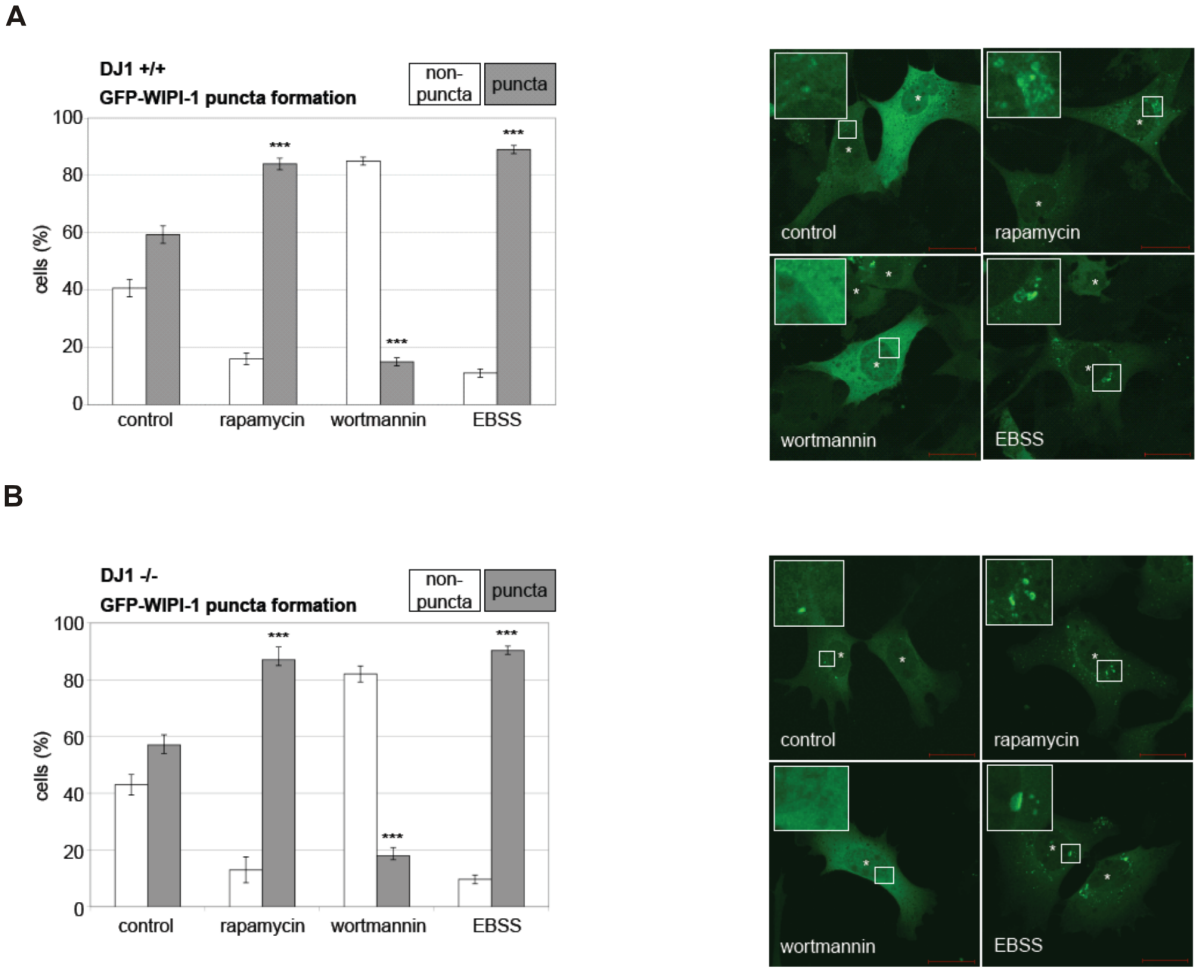
Assessing the induction and inhibition of autophagy by WIPI-1 puncta formation analyses in DJ-1 WT and DJ-1 deficient MEF. GFP-WIPI-1 puncta-formation analyses reveals that forced modulation of the autophagic activity is possible in DJ-1 deficient cells. WT (MEF DJ-1 +/+) (A) or DJ-1 KO MEF (MEF DJ-1 −/−) (B) were treated with standard inducers of autophagy, rapamycin and EBSS (medium lacking amino acids), a standard inhibitor of autophagy, wortmannin, or left untreated (mock treatment, control). Quantitative confocal microscopy was conducted. MEFs that displayed GFP-WIPI-1 puncta were counted as positive (puncta), and MEFs that did not displayed GFP-WIPI-1 were counted as negative (non-puncta). In total 200–300 MEFs were counted from 3 independent experiments (see supplemental figure S2A-H, supplemental table Table S1). *P-values* for puncta-positive DJ-1 WT cells upon rapamycin ( = 0,00067), wortmannin ( = 0,00026), EBSS ( = 0,00083), and *p-values* for puncta-positive for DJ-1 definient cells upon rapamycin ( = 0,00111), wortmannin ( = 0,00138), EBSS ( = 0,00117) are indicated (***).

In support, by measuring the induction of autophagy by human GFP-WIPI-1 puncta formation (here: transient GFP-WIPI-1 puncta formation) we demonstrate that autophagy is not principially inhibited by the loss of DJ-1 (Fig. 6A and B). No difference in the amount of GFP-WIPI-1 puncta positive cells as a measure for autophagic activity was observed in either of the standard treatments that modulate autophagy (EBSS and rapamycin as autophagy inducing agents, wortmannin as an autophagy inhibiting agent; supplemental figure S2A-H, supplemental table Table S1). From this we conclude that loss of DJ-1 results in reduced basal autophagy (LC3 protein monitoring) but not in a complete block of the autophagic capacity (WIPI-1 puncta formation, LC3 protein monitoring).

### Reduced Colocalization of Mitochondria with Lysosomes in Cellular Models of Loss of DJ-1

**Figure 7:**
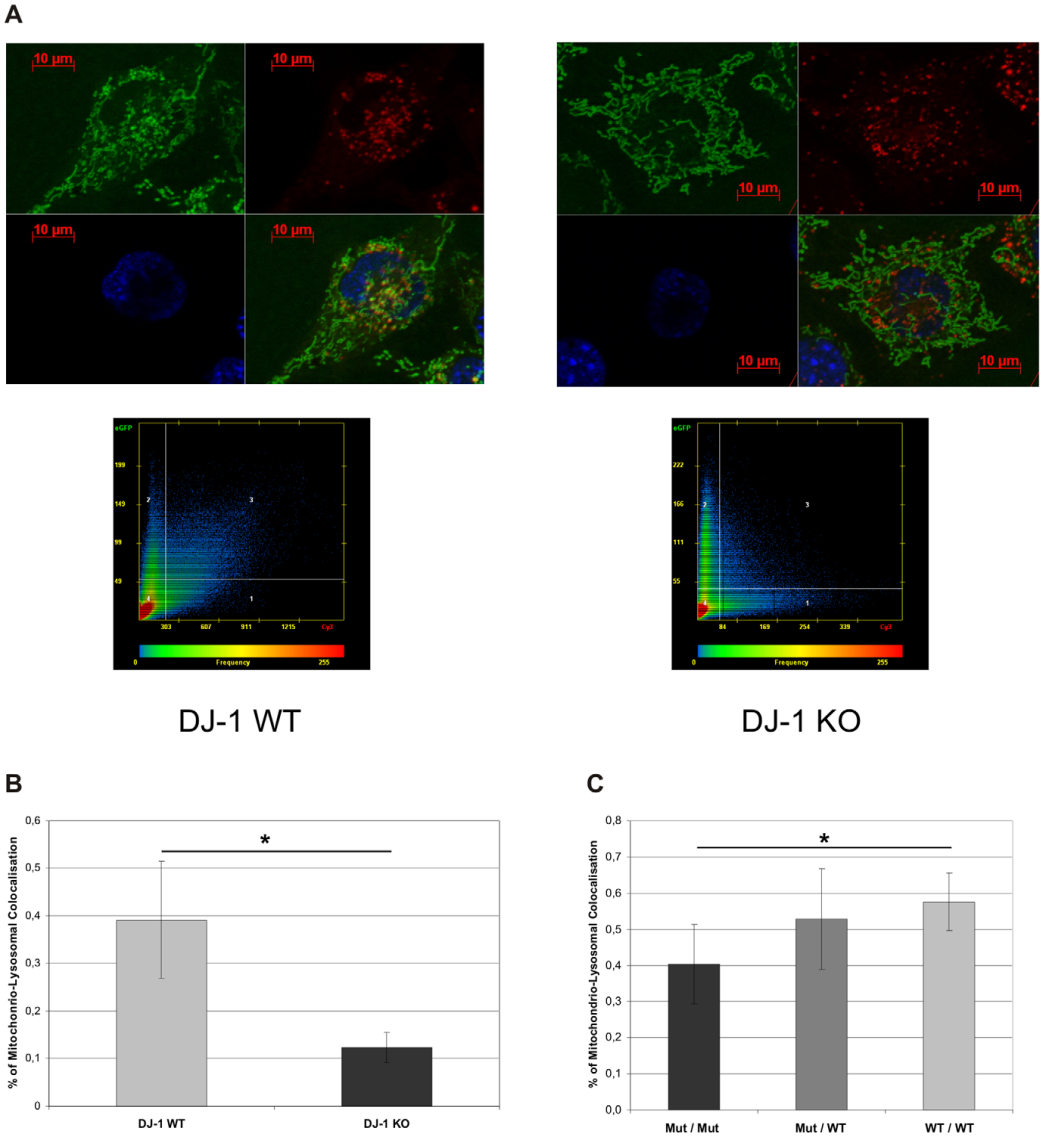
Mitochondrial and lysosomal colocalization. (A) To analyze mitochondrial and lysosomal colocalization DJ-1 KO and DJ-1 WT MEF were treated with EBSS medium and stained with 200 nM MitoTracker green and 200 nM LysoTracker red for 15 min at 37°C. Pictures were taken by life cell imaging microscope (Cell Observer Z1, Zeiss, Germany) at 37°C using ApoTome® optical slides with 0.35–0.40 z-stacks and colocalization was determined with AxioVision4.7 software (Zeiss, Germany). (B) Statistical evaluation of the scatter-plot analysis of MitoTracker green and LsoTracker red co-staining (AxioVision4.7 software, Zeiss, Germany). The experiment was performed in duplicate analysing a total of 40 cells for each cell line (*p<0.001, Student's t-test). (C) Fibroblasts from carriers of the E64D family and a healthy control were analyzed by live cell imaging using the same protocol for mitochondrial and lysosomal staining as described for MEF cells. Statistical evaluation mitochondria colocalizing with lysosomes compared to total mitochondria. We observed significantly reduced proportion of mitochondria colocalizing with lysosomes in the homozygous mutation carrier compared to healthy control (*p<0.01, Student's t-test). No significant difference was observed between heterozygous carriers and control. The experiment was performed in duplicate analyzing a total more than 30 individual cells for each proband.

Based on our finding of reduced levels of basal autophagy, we next investigated the clearance of dysfunctional mitochondria by autophagy using fluorescence microscopy on mitochondria and lysosomes. To determine the colocalization of mitochondria and lysosomes as a measure of lysosomal degradation of mitochondria we used specific dyes for differential staining of the respective organelles. Then we analyzed DJ-1 KO and DJ-1 WT MEF treated with EBSS Medium (Sigma-Aldrich, Germany) with live cell imaging (Fig. 7A). The scatter-plot analyses of colocalization pictures (Zeiss AxioVision 4.7 software, Zeiss, Germany) showed a significantly reduced co-staining of MitoTracker® green and LysoTracker® red in DJ-1 KO MEF, suggesting a reduced clearance of mitochondria by lysosomes (Figure 7A, 7B). This further supports our results showing impaired lysosomal degradation in DJ-1 KO MEF.

Next we assessed the mitochondrio-lysosomal colocalization in human fibroblasts from the E64D patient and controls. We found a significantly reduced number of mitochondria that overlap with lysosomes in EBSS-treated fibroblasts from the E64D patient compared to the healthy control (Fig. 7C). For the heterozygous mutation carriers no significant alterations were observed (Fig. 7C). Our results support the concept of reduced mitochondrial clearance due to loss of DJ-1 in mammalian cells.

### Increased Mitochondrial Mass in DJ-1 KO Cells

**Figure 8:**
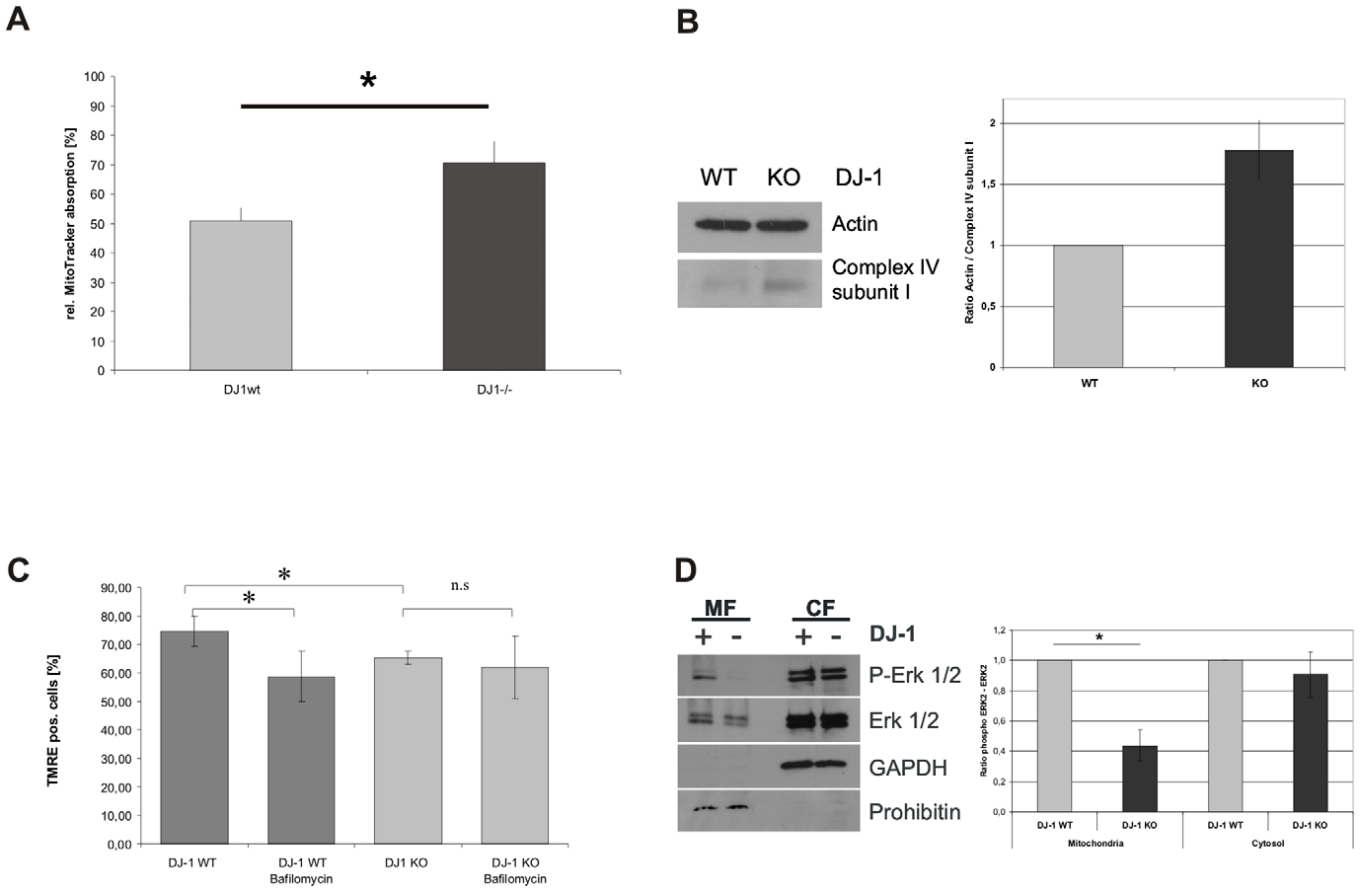
Influence of lysosomal degradation of mitochondria on mitochondrial mass and its mechanistic backround. (A) To analyze mitochondrial mass in DJ-1 KO and DJ-1 WT MEF, cells were treated with 1 µM MitoTracker® green FM (Invitrogen, USA) for 15 min at 37°C. Absorption of MitoTracker® green FM was determined by FACS analysis. The uptake of MitoTracker® was used as an indicator for the mitochondrial mass. We found evidence for increased mitochondrial mass in DJ-1 KO cells compared to controls (*p<0.001, Student's t-test). (B) To further validate our observations on mitochondrial mass, we used an independent method quantifying complex IV subunit I as a mitochondrially encoded protein of the respiratory chain. We found increased protein levels of complex IV subunit I in KO cells compared to controls as indicative of an increased mitochondrial mass in the KO condition. (C) The influence of disturbed lysosomal activity on mitochondrial function was analyzed by measuring the MMP after treatment with BafA1 as an inhibitor of lysosomal function. Cells were treated overnight with 200 nM BafA1 (Calbiochem, Germany) and the integrity of MMP was determined by measuring the uptake of TMRE (Invitrogen, USA) by FACS analysis (CyAnADP, Beckman Coulter). (C) To analyze candidate pathways for DJ-1-mediated modulation of lysosomal degradation we investigated p42/p44 MAPK (ERK1/2) phosphorylation in the mitochondrial (MF) and cytosolic fraction (CF) of DJ-1 WT and DJ-1 KO MEF. Phosphorylation and expression of the ERK1/2 kinase was analyzed by WB using antibodies against phospho-ERK1/2 (Thr202/Tyr204). To define the fractions we used antibodies against prohibitin or GAPDH, as markers for the mitochondrial and the cytosolic fraction, respectively. Quantification of relative protein levels was performed by Image J software. The results indicate a significantly reduced level of phospho-ERK2 in the mitochondrial fraction (p<0.05; Student's t test).

The regulation of mitochondrial turnover including clearance of dysfunctional mitochondria is essential for cellular homeostasis and survival [37]. Since our previous results gave evidence for disturbed mitochondrial degradation in DJ-1 KO MEF, we investigated, whether mitochondrial turnover may be affected and monitored the mitochondrial mass in cells with or without expression of physiological DJ-1. Using MitoTracker green (Molecular Probes, USA), which labels mitochondria irrespective of their functional status, we found that DJ-1 KO cells showed a significantly higher fluorescent signal than WT controls (Fig. 8A). This is indicative for an increased mitochondrial mass in DJ-1-KO cells compared to controls and correlates with our findings on lysosomal function and colocalization of mitochondria with lysosomal structures. In order to further validate our FACS-based method to quantify mitochondrial mass, we performed Western blot analyses of the mitochondrial complex IV subunit I, to quantify mitochondrial mass and to validate our FACS-based experiments for quantification by MitroTracker green staining (Invitrogen). These results showed increased levels of complex IV subunit I in DJ-1 KO cells compared to WT controls and provide additional evidence for reduced mitochondrial clearance due to loss of DJ-1 function

### Decreased MMP in DJ-1 WT Cells after Lysosomal Inhibition

To investigate whether impaired lysosomal degradation in cells expressing physiological DJ-1 is sufficient to cause impaired mitochondrial function, we treated DJ-1 WT and DJ-1 KO cells with the lysosomal inhibitor bafilomycin A1 (BafA1). BafA1 is a macrolide antibiotic that specifically inhibits vacuolar ATPase (V-ATPase) and therefore inhibits the acidification of lysosomes and promotes the accumulation of autophagosomes [38], [39]. We found that lysosomal inhibition in MEF expressing physiological DJ-1 led to similar changes in MMP as observed in untreated DJ-1 KO cells (Figure 8B). This correlates with reduced lysosomal activity and reduced basal autophagy observed in untreated DJ-1 KO cells (Figure 4A and Figure 5A–C). Interestingly no further decrease in MMP was observed in DJ-1 KO cells upon treatment with BafA1 (Fig. 8B).

### Analysis of Mechanisms of Mitophagy

Regulation of autophagy and especially the autophagic degradation of mitochondria is an essential tool to maintain mitochondrial function and cellular homeostasis. Recent studies showed that DJ-1 is involved in the regulation of extracellular signal-regulated protein kinase 1/2 (ERK1/2) and ERK1/2 was shown to be necessary and sufficient to induce macroautophagy and mitochondrial turnover [40]. Since mitochondrially localized ERK2 was described to be involved in the regulation of macroautophagy [41], we investigated, whether differences in the levels of activated cytoplasmic and mitochondrial ERK1/2 may contribute to the observed accumulation of dysfunctional mitochondria. Our results show a reduction in phosphorylated ERK2 in the mitochondrial fraction of DJ-1 KO MEF, while there was no difference in the phosphorylation of neither ERK1 nor ERK2 in the cytosolic fraction between DJ-1 KO and DJ-1 WT MEF. These results support a possible involvement of DJ-1 mediated ERK2 phosphorylation in the control of autophagic and lysosomal function.

## Discussion

Mitochondria have been early recognized as a critical interface between vital energy metabolism and cell death. Based on biochemical findings in affected brain regions of PD patients a role of mitochondrial dysfunction and oxidative damage has been hypothesized in both ageing and neurodegeneration [42]. This hypothesis was further emphazised by the identification of nuclear genes mutated in familial forms of PD that are directly linked to mitochondrial function, i.e. Parkin, PINK1 and DJ-1 [10], [43], [44]. In the present study we demonstrate, that the Parkinson's disease associated protein DJ-1 integrates both mitochondrial and lysosomal function.

We have shown that the loss of the mitochondrial protein DJ-1 KO in MEF cells causes multiple mitochondrial impairments including functional and morphological changes: (i) a significant increase of mitochondrial ROS formation in DJ-1 KO cells which could impair the oxidative phosphorylation as indicated by (ii) diminished pyruvate dependent respiration rates and (iii). reduced MMP. Importantly, the decreased MMP as a parameter of mitochondrial dysfunction was restored by complementation of the DJ-1 KO model with physiological active DJ-1 protein (Fig. 2C). These metabolically detectable impairments were paralleled by morphological changes as decreased mitochondrial branching which could be explained by a relative augmentation of fission events. Since there was no change in the steady state levels of the main fusion/fission mediators Drp1, Mfn2, Fis1, and OPA1 (Fig. 3C), we speculate that increased fission could be caused by a reduced energy supply to the energy consuming fusion processes. Indeed regarding our findings on mitochondrial depolarization these results support the notion that relevant reduction in the MMP negatively regulates the prevalence of fusion events (Twig et al., 2008). Disrupted fusion results in mitochondrial heterogeneity and decreased rates of cellular respiration was recently shown in cellular models of null mutations of Mfn1 and Mfn2 [45]. Moreover it seems that cells with impaired fusion/fission dynamics are unable to use mitochondrial redistribution in order to provide increased local ATP supply at cellular sites of particular demand [46].

Loss of DJ-1 shifted the mitochondrial network towards the status of reduced mitochondrial connectivity. Indeed our first study of fibroblasts derived from patients with homozygous mutations in the *DJ-1* gene revealed similar phenotype with reduced mitochondrial branching in the human condition (Fig. 3D, 3E) indicating that DJ-1 may interfere with the critical balance between mitochondrial fusion and fission dynamics.

First evidence for a potential role of DJ-1 in the regulation of mitochondrial morphology came from comparative studies of different generated DJ-1 mutants that influenced the oxidative modification of a critical cysteine in position 106 [11]. In the same study, functional mitochondrial connectivity was assayed in DJ-1 KO MEF by fluorescence recovery after photobleaching (FRAP) measurement and indicated a loss of connectivity. This is consistent with our present study using direct morphometry as an independent method to assess mitochondrial connectivity.

Recently, effects on mitochondrial morphology and a critical role for mitochondrial homeostasis in PD has been attributed to the loss of PINK1 function, and it was suggested that the PD-associated proteins PINK1 and Parkin may act in the same pathway to modulate mitochondrial morphology [44], [47], [48]. In mammalian cells, loss of PINK1 led to increased fission events causing mitochondrial fragmentation, that were rescued by Parkin [44], [49]. However, in invertebrate *in vivo* models loss of PINK1 function increased mitochondrial fusion events, that was rescued by increasing Drp1 gene dosage [47]. Thus species-specific differences in the regulation of mitochondrial dynamics may exist and may explain the variable deflection of the critical balance between fusion and fission events in different *in vivo* and *in vitro* models. In this context it is of note that we observed a similar phenotype in a murine and human cellular model of loss of DJ-1 function. Whether the mitochondrial phenotype caused by loss of DJ-1 (our study here) and PINK1 function reflects converging signaling pathways warrants further studies. However, previous data of our own research as well as *in vivo* studies in Drosophila models of PINK1 inactivation argue against a functional compensation of PINK1 by DJ-1 [44], [50].

Mitochondrial integrity is critically secured by autophagic clearance [51], [52]. During the process of autophagy unique vesicles, autophagosomes, are generated that sequester the cytoplasmic cargo, including cellular organelles and long-lived proteins. Subsequently, autophagosomes fuse with lysosomes for cargo degradation. Autophagy occurs on a constitutive level in all eukaryotic cells and as a consequence, cellular homeostasis is secured by constant clearance and recycling of cytoplasmic constituents (basal autophagy). Importantly, due to a variety of cellular insults, including ROS production, autophagy is induced to an extended degree (induced autophagy) [32], [53]. In this respect, ROS of mitochondrial origin, depolarization of mitochondria and increased fission events each have been reported to trigger autophagic activity [41], [54]. However, although we measured an increase of mitochondrial ROS production, a prominent depolarization of mitochondria in DJ-1 KO cells, and reduced mitochondrial connectivity, the basal autophagy levels in these cells were reduced compared to WT controls (Fig. 5).

Autophagic pathways are an important protection system against ROS-mediated damage of proteins and organelles in the cell [52]. These pathways are compromised during aging and age-related disorders like PD are characterized by the accumulation of oxidized proteins and dysfunctional mitochondria [4], [55], [56], [57]. In support, premature aging, that is hypothesized to be elementary connected to misregulated autophagy [58], represents a known mechanism involved in PD pathogenesis [59]. Importantly, loss of key autophagy proteins, such as Atg5 have been shown to be responsible for reducing basal autophagy levels that led to neurodegenerative symptoms in mouse models [60]. In Atg5-deficient peripheral T cells reduced levels of basal autophagy lead to an impaired removal of damaged mitochondria that resulted in increased mitochondrial mass [61]. Indeed we demonstrated that human and murine cells lacking physiological DJ-1 show evidence for a decrease in mitochondrial degradation (Fig. 7) and an increase in mitochondrial mass (Fig. 8A). In further support, the multilamellar lysosomal structures accumulating in DJ-1 KO cells are highly reminiscent of pigmented vacuoles that represent autophagic responses due to neurodegeneration and support the idea of a mitochondrial-lysosomal axis of aging [31].

Recent studies provided first evidence for a potential role of DJ-1 in autophagic response to cellular stress. In human osteosarcoma cells an impairment of hypoxia-induced autophagy after DJ-1 knock-down was described by analyzing LC3 [62]. Moreover, a role of DJ-1 in the paradigm of paraquat-induced autophagy was defined in a knock-down model of DJ-1 in human neuroblastoma cells [63]. However, effects on basal autophagy as demonstrated in our DJ-1 KO cellular model, were not described in both siRNA knock-down models, most probably due to the remaining DJ-1 function mediated by residual protein levels of endogenous DJ-1 after siRNA treatment [62], [63]. In our model further induction of autophagy by amino acid deprivation or rapamycin treatment showed the presence of autophagic flux in both, DJ-1 KO and WT cells. However levels of autophagic activity remained reduced for the DJ-1 KO condition compared to control cells as demonstrated by LC3 protein monitoring.

Based on our findings of a functional role of DJ-1 in preserving basal autophagy the observed changes in mitochondrial connectivity due to loss of DJ-1 may be secondary to the accumulation of damaged mitochondria. Indeed, lysosomal storage disorders are characterized by an inefficient autophagic clearence of mitochondria leading to loss in mitochondrial connectivity [64]. Mitochondrial alterations observed in lysosomal storage disorders were replicable by treatment of control cells with lysosomal inhibitors (Jennings et al., 2006). We also found that lysosomal inhibition of control MEF expressing physiological DJ-1 led to similar changes in MMP as observed in DJ-1 KO cells (Fig. 8B).

In order to understand the connection between ROS production and autophagy levels in more detail, it will be of great interest to identify signaling cascades by which ROS triggers autophagy [65]. Here we provide evidence for a correlation of DJ-1 protein function and ERK1/2 signaling pathways that are involved in the regulation of autophagy. Recent studies demonstrated an involvement of ERK2 in the regulation of autophagy [41], [66]. It has been shown that ERK1/2 is involved in the modulation of lysosomal degradation and that basal levels of ERK2 kinase activity are required for its effect on autophagy [67]. In this context higher levels of phopho-ERK2 and/or mitochondrial ERK2 localization induced autophagy including mitochondrial clearance [41]. Consistently, we found that DJ-1 KO cells display both, a decrease in mitochondrial ERK2 activation and basal autophagy (Fig. 5 and Fig. 8C). In further support, the neuroprotective role of DJ-1 in terms of oxidative stress was recently found to be related to the activation of the ERK1/2 signaling pathway [40].

Based on previous findings regarding the function of the DJ-1 protein and our present study, we suggest that DJ-1 might function in regulating cellular organelle homeostasis. Upon mitochondrial ROS production, DJ-1 quenches ROS levels [10], [11] thereby preventing cell death and preserving the integrity of mitochondria and lysosomes. Our findings suggest that loss of DJ-1 leads to the accumulation of dysfunctional mitochondria characterized by increased intramitochondrial ROS levels and reduced MMP that under physiological conditions would be compensated by increased housekeeping-degradation of damaged organelles via autophagy. However, due to disturbed autophagic clearance of damaged mitochondria, as demonstrated by lysosomal alterations that include a reduction of lysosomal activity and of basal auophagy levels, and the appearance of multilamellar lysosome-like intracellular bodies, mitochondrial dynamics shift to reduced connectivity. On this basis it will be of great interest to define the regulative signaling cascades, such as the ERK pathway, that modulate the function of DJ-1 in maintaining mitochondria and lysosomal homeostasis.

## Supporting Information

Figure S1 Protein levels of DJ-1 in fibroblasts from carriers of the E64D mutation in the *DJ-1* gene and a healthy control. The pedigree of the E64D family shows one affected patient carrying a homozyous E64D mutation (dark grey sign) and two sibling that carry the mutation in the heterozygous state (light grey sign). Western blot analyses using an antibody against human DJ-1 revealed markedly reduced levels of endogenous DJ-1 in the homozygous carrier (Mut/Mut) of the E64D mutation. Physiological levels of DJ-1 are represented by an unrelated healthy control (WT/WT; white sign). Heterozygous carriers (Mut/WT) were unaffected and display similar levels of DJ-1 protein as the control.
(0.18 MB TIF)

Figure S2 GFP-WIPI-1 puncta-formation analyzes reveals an unaltered induction of autophagy in DJ-1 KO MEF. Quantification (Supplemental Table S1) of confocal microscopy (Supplemental Figures S2 A-H) of GFP-WIPI-1 protein puncta as a measure for the onset of autophagy was conducted from three independent sets of experiments (100 cells in each of the experiments) in WT and DJ-1 KO MEF.
(2.53 MB TIF)

Table S1 GFP-WIPI-1 puncta-formation analyzes reveals an unaltered induction of autophagy in DJ-1 KO MEF. Quantification (Supplemental Table S1) of confocal microscopy (Supplemental Figures S2 A-H) of GFP-WIPI-1 protein puncta as a measure for the onset of autophagy was conducted from three independent sets of experiments (100 cells in each of the experiments) in WT and DJ-1 KO MEF.
(0.66 MB TIF)
