# Supplementary figures and images for: Reduced Basal Autophagy and Impaired Mitochondrial Dynamics Due to Loss of Parkinson's Disease-Associated Protein DJ-1
Source: PLoS One. 2010 Feb 23;5(2):e9367. doi: 10.1371/journal.pone.0009367 (PMC2826413; doi:10.1371/journal.pone.0009367)

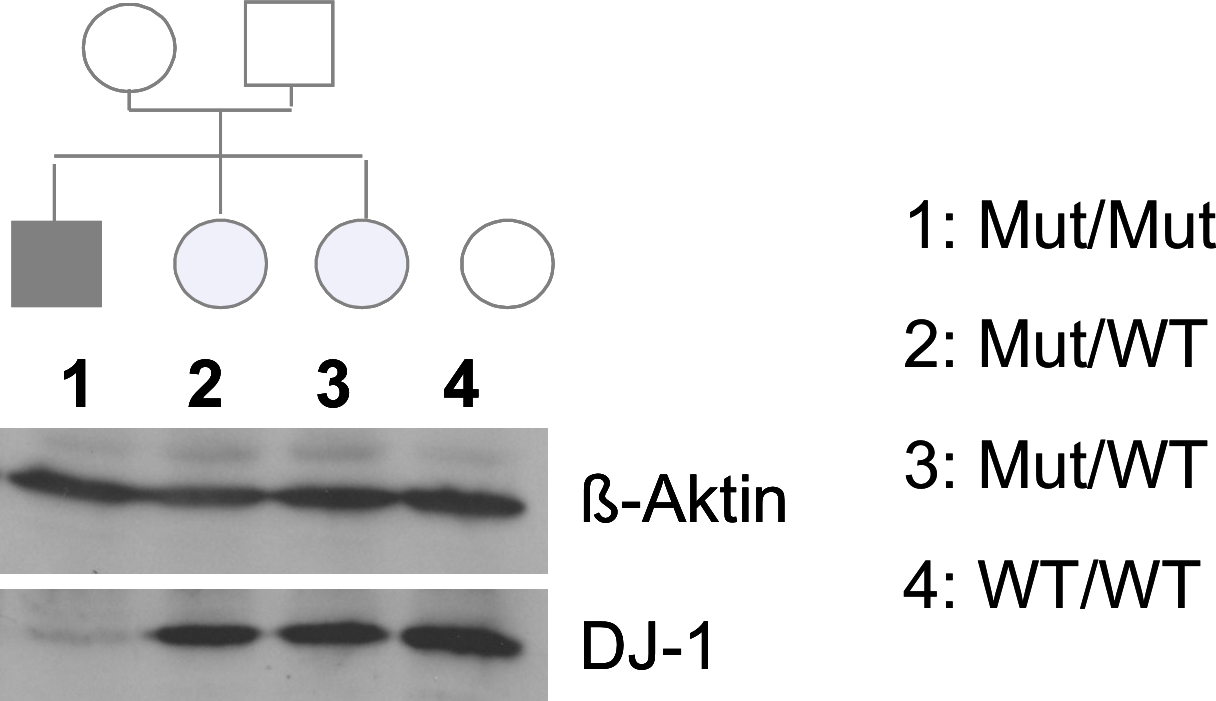

Supplement: Figure S1 — Protein levels of DJ-1 in fibroblasts from carriers of the E64D mutation in the DJ-1 gene and a healthy control. The pedigree of the E64D family shows one affected patient carrying a homozyous E64D mutation (dark grey sign) and two sibling that carry the mutation in the heterozygous state (light grey sign). Western blot analyses using an antibody against human DJ-1 revealed markedly reduced levels of endogenous DJ-1 in the homozygous carrier (Mut/Mut) of the E64D mutation. Physiological levels of DJ-1 are represented by an unrelated healthy control (WT/WT; white sign). Heterozygous carriers (Mut/WT) were unaffected and display similar levels of DJ-1 protein as the control. (0.18 MB TIF) [file pone.0009367.s001.tif]

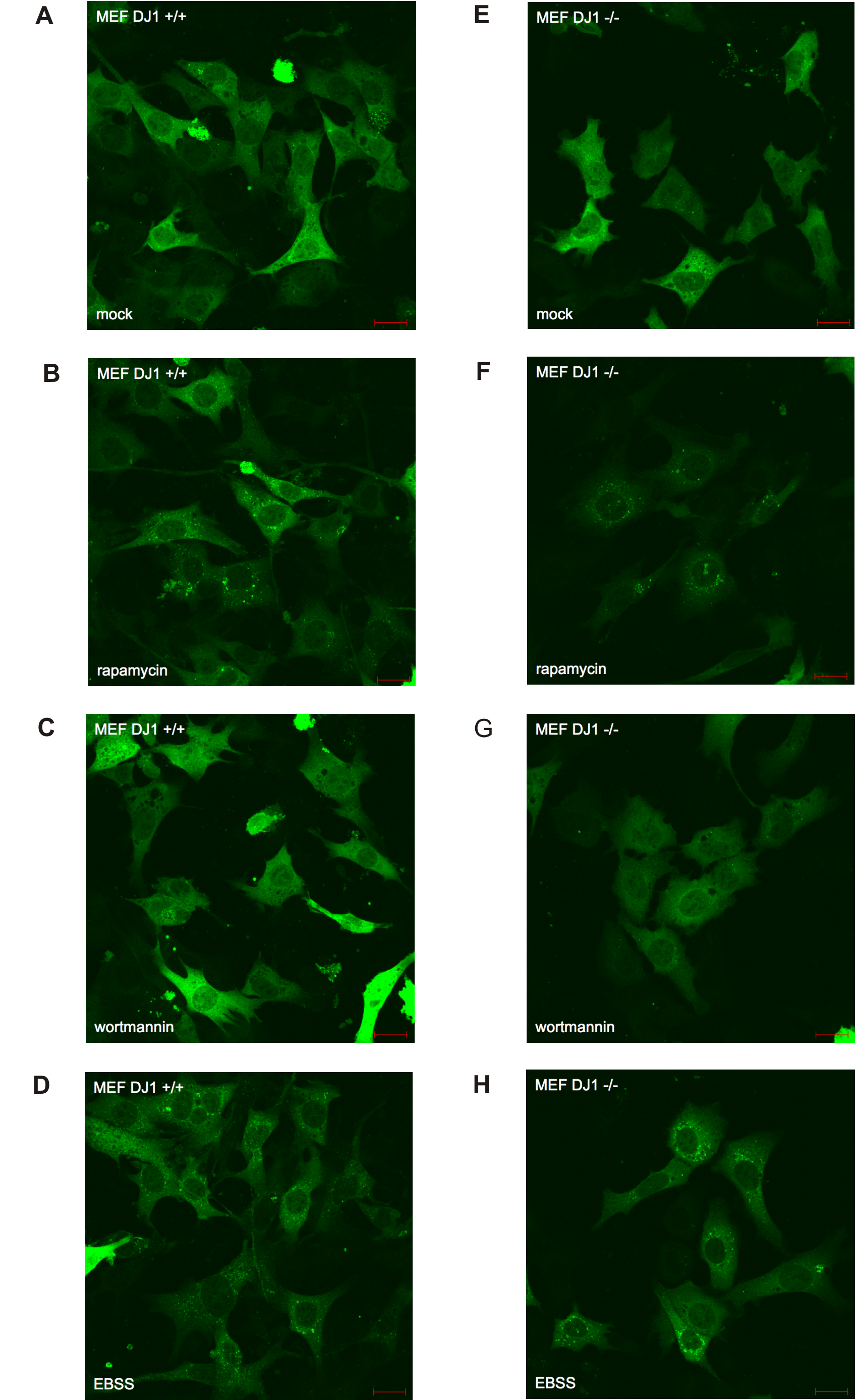

Supplement: Figure S2 — GFP-WIPI-1 puncta-formation analyzes reveals an unaltered induction of autophagy in DJ-1 KO MEF. Quantification (Supplemental Table S1) of confocal microscopy (Supplemental Figures S2 A-H) of GFP-WIPI-1 protein puncta as a measure for the onset of autophagy was conducted from three independent sets of experiments (100 cells in each of the experiments) in WT and DJ-1 KO MEF. (2.53 MB TIF) [file pone.0009367.s002.tif]

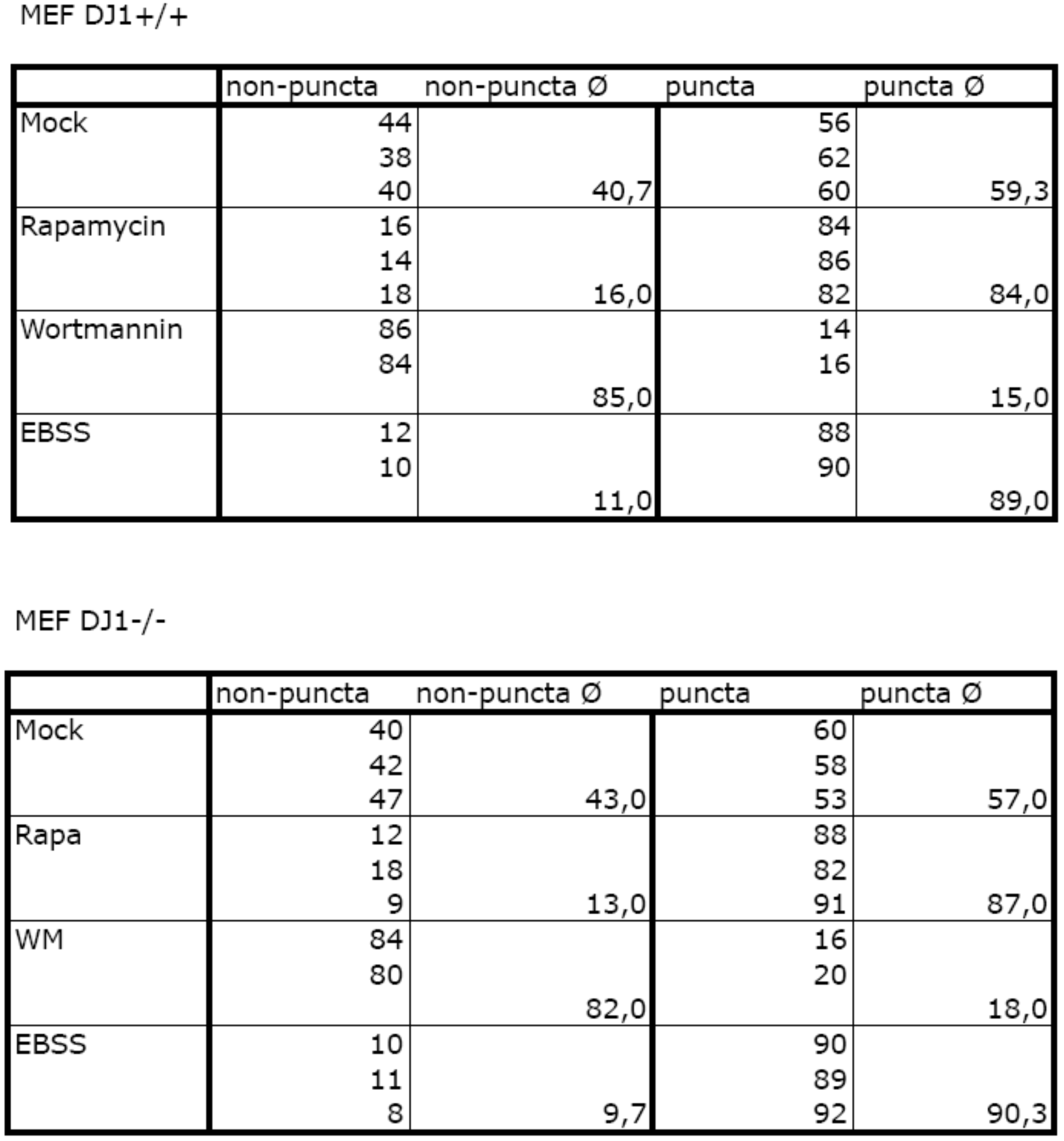

Supplement: Table S1 — GFP-WIPI-1 puncta-formation analyzes reveals an unaltered induction of autophagy in DJ-1 KO MEF. Quantification (Supplemental Table S1) of confocal microscopy (Supplemental Figures S2 A-H) of GFP-WIPI-1 protein puncta as a measure for the onset of autophagy was conducted from three independent sets of experiments (100 cells in each of the experiments) in WT and DJ-1 KO MEF. (0.66 MB TIF) [file pone.0009367.s003.tif]
